# An improved medium formulation for efficient *ex vivo* gene editing, expansion and engraftment of hematopoietic stem and progenitor cells

**DOI:** 10.1016/j.omtm.2023.02.014

**Published:** 2023-02-28

**Authors:** Rajeev Rai, Asma Naseem, Winston Vetharoy, Zohar Steinberg, Adrian J. Thrasher, Giorgia Santilli, Alessia Cavazza

**Affiliations:** 1 Infection, Immunity and Inflammation Research and Teaching Department, Great Ormond Street Institute of Child Health, University College London, 30 Guilford Street, London WC1N 1EH, UK

**Keywords:** gene editing, hematopoietic stem cells, cell culture, culture medium

## Abstract

Gene editing has emerged as a powerful tool for the therapeutic correction of monogenic diseases. CRISPR-Cas9 applied to hematopoietic stem and progenitor cells (HSPCs) has shown great promise in proof-of-principle preclinical studies to treat hematological disorders, and clinical trials using these tools are now under way. Nonetheless, there remain important challenges that need to be addressed, such as the efficiency of targeting primitive, long-term repopulating HSPCs and their *in vitro* expansion for clinical application. In this study, we assessed the effect of different culture medium compositions on the ability of HSPCs to proliferate and undergo homology-directed repair-mediated knock-in of a reporter gene, while preserving their stemness features during *ex vivo* culture. We demonstrated that by supplementing the culture medium with stem cell agonists and by fine-tuning its cytokine composition it is possible to achieve high levels of gene targeting in long-term repopulating HSPCs both *in vitro* and *in vivo*, with a beneficial balance between preservation of stemness and cell expansion. Overall, the implementation of this optimized *ex vivo* HSPC culture protocol can improve the efficacy, feasibility, and applicability of gene editing as a key step to unlocking the full therapeutic potential of this powerful technology.

## Graphical abstract

**Figure:**
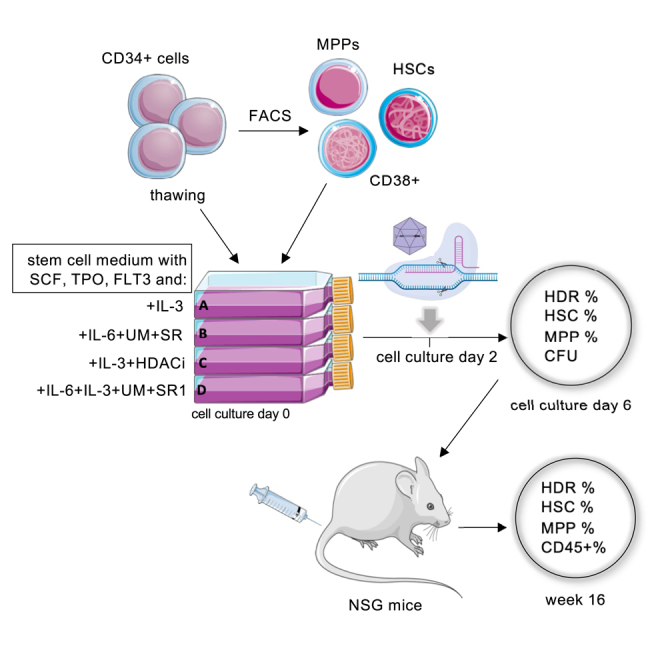

## Teaser

Hematopoietic stem cell (HSC) gene editing faces important challenges, including targeting of primitive HSCs and their expansion for clinical application. Rai et al. assess the influence of *ex vivo* culture conditions on HSC stemness and targetability, showing that fine-tuning of medium components significantly improves the feasibility of therapeutic HSC gene editing.

## Introduction

Over the past 2 decades, autologous hematopoietic stem and progenitor cell (HSPC) genetic engineering has been successfully applied to the treatment of blood monogenic disorders through an *ex vivo* process by which HSPCs are isolated from the patient, genetically modified, and then reinfused back to the patient where they engraft and differentiate, establishing a population of functionally corrected blood cells. Although there are several tools able to mediate gene transfer to target cells, the CRISPR-Cas9 system has recently proven to be a versatile platform for gene addition and deletion strategies in the arena of blood disorders, and ongoing clinical trials on HSPC gene editing to treat hemoglobinopathies are showing encouraging results.^1^^,^^2^ The CRISPR-Cas9 platform relies on a DNA-binding guide RNA (gRNA), which is complementary to the target DNA sequence, and a Cas9 endonuclease that creates a double-strand break upon binding to the target site on the DNA, triggering the activation of two main endogenous repair pathways: non-homologous end-joining (NHEJ) and homology-directed repair (HDR). Each of these pathways could be exploited for therapeutic purposes. In particular, HDR mediates the accurate repair of the cut by using a DNA sequence homologous to the region flanking the double-strand break as a template. As this process results in the insertion of a correct DNA sequence at the target site, this pathway can be harnessed to treat those diseases for which correcting or adding a genetic element may lead to a therapeutic benefit, mimicking the gene addition approach achieved through viral gene therapy. High frequency of HDR-mediated gene insertion has been successfully obtained in HSPCs by our team^3^ and others^4^; however, achieving complete disease correction by means of gene editing remains cumbersome and there are still many challenges facing the field, including the specificity and efficiency of the editing system *in vivo*.

For the successful treatment of blood disorders, both long-term repopulating multipotent hematopoietic stem cells (HSCs) and far more committed progenitor cells with limited self-renewing and repopulating capacity must be genetically modified, to allow long-term correction of hematopoiesis and rapid blood reconstitution, respectively.^5^ Although to different extents, both viral gene therapy and HDR-based gene editing approaches have shown preferential genetic correction of more abundant, proliferating progenitors, rather than of rarer, more quiescent HSCs, when manipulating CD34+ HSPCs *ex vivo.*^6^^,^^7^ For gene editing approaches, the lower frequency of gene correction by HDR in HSCs is likely caused by a combination of different factors, including the inefficient delivery of the donor template by viral transduction, the quiescent nature of HSCs, which reduces the activity of the HDR pathway,^8^ and the intrinsic cytotoxicity of the procedure. Several strategies have been adopted to increase gene editing efficiency in these cells. Transient manipulation of the DNA repair pathways, favoring HDR over NHEJ for example, has been tried as a means to increase the frequency of gene knock-in, with only limited improvements in HSPCs.^9^^,^^10^ The most successful approach so far has probably arisen from the attempt to promote HSC cell-cycle progression to increase the engagement of HDR components and hence the knock-in of exogenous sequences, which has been accomplished by the use of cell-cycle modulators.^11^^,^^12^ Overall, the implementation of these methods, including optimized timing and dosage of the editing reagents^13^^,^^14^^,^^15^ to circumvent gene editing limitations have led to relatively high levels of gene engineering in HSPCs (up to 80%) and, to a lesser extent, in HSCs (up to 50%) *in vitro* and *in vivo*, with correction rates matching those frequently obtained by viral gene addition strategies and predicted to be curative for many inherited blood disorders.^3^^,^^9^^,^^16^^,^^17^^,^^18^

Despite the excellent levels of correction currently attainable *in vitro,* animals transplanted with *ex vivo* manipulated HSPCs display an overall lower human engraftment rate than those infused with unmanipulated control cells, and more importantly a decrease in the frequency of engrafted corrected cells over the course of the 16–30 weeks after transplantation when studied in immunodeficient mice. There is evidence that reduction of the frequency of corrected cells *in vivo* is negligible early after transplantation, but becomes prominent at later stages, suggesting that the cause lies within the population of HSCs as an effect of either their limited correction, limited presence in the population infused, or their limited engraftment and self-renewing ability after manipulation.^19^ Therefore, strategies to preserve HSCs during the *ex vivo* manufacturing process are required, to ensure their abundance in the infused cellular product and the maintenance of their long-term repopulating characteristics *in vivo*. Indeed, it is well known that prolonged cell culture (>48 h) and stimulation protocols, usually adopted for efficient gene transfer in HSPCs, can exert a detrimental effect on their engraftment and long-term repopulation capacity.^20^^,^^21^^,^^22^^,^^23^ Recent findings showed that addition to the culture medium of compounds proposed to preserve stemness, such as Stem Regenin-1 (SR-1), UM171, and 16,16-dimethyl prostaglandin E2 (dmPGE2), helps to maintain the long-term multilineage repopulation capacity of human corrected HSPCs transplanted in immunodeficient mouse models, partially overcoming the drawbacks of prolonged culture.^13^^,^^24^^,^^25^^,^^26^

In this study, we sought to evaluate existing and newly defined HSPC culture conditions based on their ability to support gene targeting of primitive HSCs while favoring their expansion *in vitro* and transplantation *in vivo*. We show that the addition of interleukin (IL)-3 to the stem cell medium greatly supports HSPC expansion and HDR-mediated transgene knock-in while promoting cell differentiation and limited maintenance of long-term repopulating HSCs. On the contrary, media containing IL-6 better preserve HSPC stemness at the expense of cell proliferation and HDR frequencies. We therefore tested different combinations of compounds and demonstrated that by supplementing the culture medium with inhibitors of histone deacetylases, and/or by fine-tuning its cytokine composition it is possible to achieve high levels of gene targeting with a beneficial balance between preservation of stemness and cell expansion *in vitro* and *in vivo*.

## Results

### Evaluating the effect of IL-3 and IL-6 on cultured HSPCs

**Figure 1:**
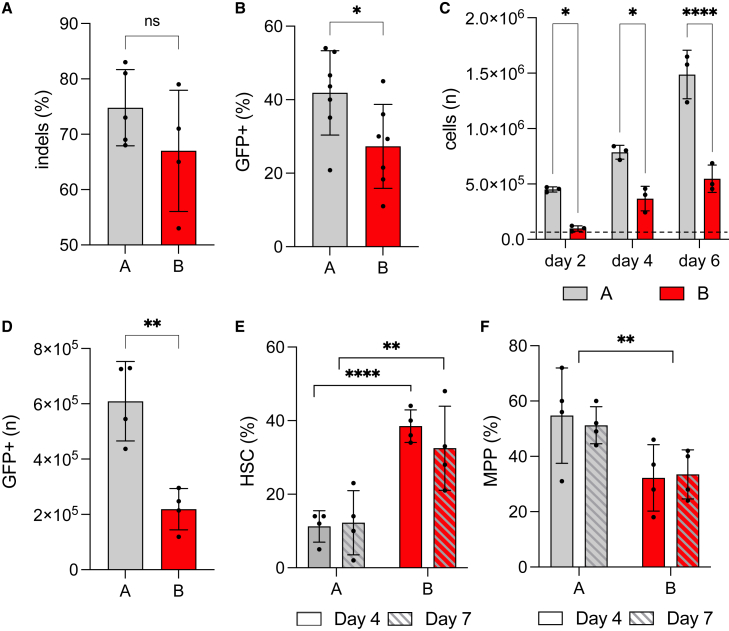
Comparison of *ex vivo* culture and manipulation of HSPCs grown in media supplemented with either IL-3 or IL-6+SR1+UM171 Frequency of (A) NHEJ-mediated repair (indels) and (B) HDR-mediated knock-in of a PGK-GFP reporter cassette at the *WAS* locus in CD34+ HSPCs cultured in medium A or medium B. (C) Total number of cells retrieved after 2, 4, and 6 days of culture in the two media. The dotted line represents the starting cell number (100,000 cells). (D) Total number of edited cells retrieved in either medium after 6 days of culture. (E) Frequency of HSCs (CD34+ CD38- CD90+ CD45RA- cells) and (F) MPPs (CD34+ CD38- CD90- CD45RA- cells) detected in the CD34+ bulk cultured in either medium 4 and 7 days after gene editing (6 and 9 days of culture, respectively). HDR, homology-directed repair; HSC, hematopoietic stem cell; MPP, multipotent progenitor; NHEJ, non-homologous end-joining. Data in Figure 1 are presented as mean ± SD, with n = 4 biological replicates; p values were calculated using one-way ANOVA with Tukey’s comparison test (E, F) or two-tailed unpaired Student’s t test (A–D) (∗p < 0.05; ∗∗p < 0.01; ∗∗∗p < 0.005; ∗∗∗∗p < 0.001; no asterisk = nonsignificant).

To assess the impact of medium composition on HSPC expansion, stemness, and efficiency of DSB repair via HDR, we used a CRISPR-Cas9 platform we previously designed to target the *WAS* locus, comprising a High-Fidelity Cas9 ribonucleoprotein complex (RNP) targeting *WAS* exon 1 and an AAV6 donor vector carrying a PGK-GFP reporter cassette to be inserted in the *WAS* locus. This platform has been chosen for its optimal performance, with consistent high levels of gene knock-in across different CD34+ HSPC donors and experimental replicates and good rates of engraftment of edited cells *in vivo* with limited toxicity.^7^ Moreover, knockout of the *WAS* gene, caused by the insertion of a PGK-GFP cassette, does not affect the function, frequency or the repopulating potential of HSPCs,^3^^,^^27^ further confirming the suitability of the platform for this study. We first focused our attention on the basal composition of the medium used to culture HSPCs with the purpose of gene manipulation. Traditionally, HSPC *ex vivo* culture protocols have provided the use of a serum-free culture medium supplemented with a predefined stem cell cocktail that includes human FMS-related tyrosine kinase 3 ligand (Flt3L), human thrombopoietin (TPO), and human stem cell factor (SCF), with the addition of basal cytokines to promote cell fitness and proliferation. HSPC viral gene therapy manufacturing has relied for a long time on the addition of 20–60 ng/mL IL-3 to the basal medium composition,^28^^,^^29^^,^^30^ more recently, replacement of IL-3 with IL-6, and especially with the further addition of stem cell agonists SR-1 and UM171 coupled with low cell density, has proven to maintain high frequency of HSCs *in vitro* and partially *in vivo*, in gene therapy as well as gene editing applications.^6^^,^^11^^,^^14^^,^^24^ As such, we first tested the impact of these two medium compositions on the ability of HSPCs to proliferate and undergo HDR-mediated knockin of a PGK-GFP reporter cassette into the *WAS* locus. To this aim, HSPCs harvested from mobilized peripheral blood (PB) of up to four different healthy donors were cultured for 2 days in a basal, FLT3-TPO-SCF-containing medium supplemented with either IL-3 or IL-6 and then electroporated with the Cas9-gRNA RNP and transduced with the AAV6 donor vector using established protocols.^3^ Cells were kept in culture for 2 additional days, after which they were harvested to assess gene editing efficiency and cell phenotype. HSPCs cultured in IL-3-containing media showed levels of NHEJ-mediated repair of the DSB induced at the *WAS* locus comparable to IL-6-containing media (Figure 1A), but with a higher frequency of HDR-mediated GFP integration, as assessed by flow cytometry and ddPCR, with high concordance between the two techniques (average 42% and 27%, respectively; Figures 1B, S1A, and S1B). As the engagement of the HDR machinery is strictly cell-cycle dependent,^8^ we assumed that the high knock-in frequency was a direct consequence of increased HSPC proliferation mediated by IL-3. To confirm this, cells were thawed and seeded in equal numbers in the two different media and cell counting was performed after 2, 4, and 6 days of culture; we indeed noticed a 4.5-, 2.1-, and 2.4-fold increase in total cell numbers, respectively, when cells were cultured in medium A vs. medium B (Figure 1C), which, combined with higher knock-in rates achieved with the first, translated into an average of 2.6-fold more GFP-positive HSPCs found in the IL-3 containing medium (Figure 1D). To investigate the ability of the media to preserve the stemness of HSPCs expanded *in vitro*, we assessed the frequency of primitive, long-term repopulating HSCs (HSCs; CD34+ CD38- CD90+ CD45RA- cells) and multipotent progenitors (MPPs; CD34+ CD38- CD90- CD45RA- cells) in the bulk CD34+ HSPC population by flow cytometry using a well-established panel of markers (Figure S1A).^31^^,^^32^^,^^33^ We found a 3.4- and a 2.7-fold reduction in the frequency of HSCs within CD34+ HSPCs cultured in the presence of IL-3 vs. IL-6+SR1+UM171, at 4 and 7 days after editing, respectively (Figure 1E); on the contrary, MPPs were better preserved by IL-3-containing medium compared with the other, albeit at a lower fold change (Figure 1F). Even though media A and B differ not only by the cytokine content, but also by the presence of stem cell agonists in the latter, the presence of IL-3 vs. IL-6 appears to be the main driver of the different effects exerted by the two medium compositions on cultured HSPCs. Indeed, when cells were cultured in basal media with TPO, FLT3L, and SCF and the sole addition of either IL-3 or IL-6, we observed similar results, with IL-3-containing medium promoting efficient HDR and increased proliferation, resulting in a 6.6-fold overall increase in the number of edited, GFP+ HSPCs compared to IL-6 medium (Figures S1C–S1E). HSPCs cultured in the IL-3 medium saw a 2-fold drop in the frequency of HSCs and a similar increase in the frequency of MPPs at all time points (Figures S1H–S1I), although the difference in HSC content was less striking than that seen in the medium A vs. medium B comparison. The data corroborate the evidence that the two stem cell agonists, SR-1 and UM171, contained in medium B indeed play a key role in the preservation of stemness; however, they appear to have no influence on the efficiency of HDR in HSPCs and HSCs (Figures S1F–S1G). Overall, our data are in line with previous studies and observations, where IL-3 has been reported to promote the proliferation of HSPCs, together with increased cell differentiation and reduction of their repopulating potential *in vivo*.^34^^,^^35^^,^^36^

### Testing new medium formulations for *ex vivo* HSPC culture and gene editing

Given the superior rates of targeted integration achieved with IL-3-supplemented media and the increased HSC content when IL-6 is added, we sought to define new medium configurations that could either (1) increase the stemness-preserving capacity of IL-3-containing media (medium A); or (2) increase the proliferation ability and thus engagement of the HDR machinery of the currently widely used IL-6 containing media (medium B^12^^,^^14^^,^^24^). To this aim, we investigated the effect of supplementing medium A with HDAC inhibitors (HDACi) (medium C), which were previously shown to successfully promote a HSC phenotype through the regulation of epigenetic plasticity and chromatin structures critical for the maintenance of the primitive status of HSCs.^37^^,^^38^^,^^39^ HDACi has the additional advantage of increasing the efficiency of gene editing in human cells,^40^^,^^41^^,^^42^ and as such were favorably chosen among the plethora of available stemness-preserving small compounds. In parallel, we also tested the addition of IL-3 to the IL-6, UM171, and SR1-containing medium B (resulting in medium D).

**Figure 2:**
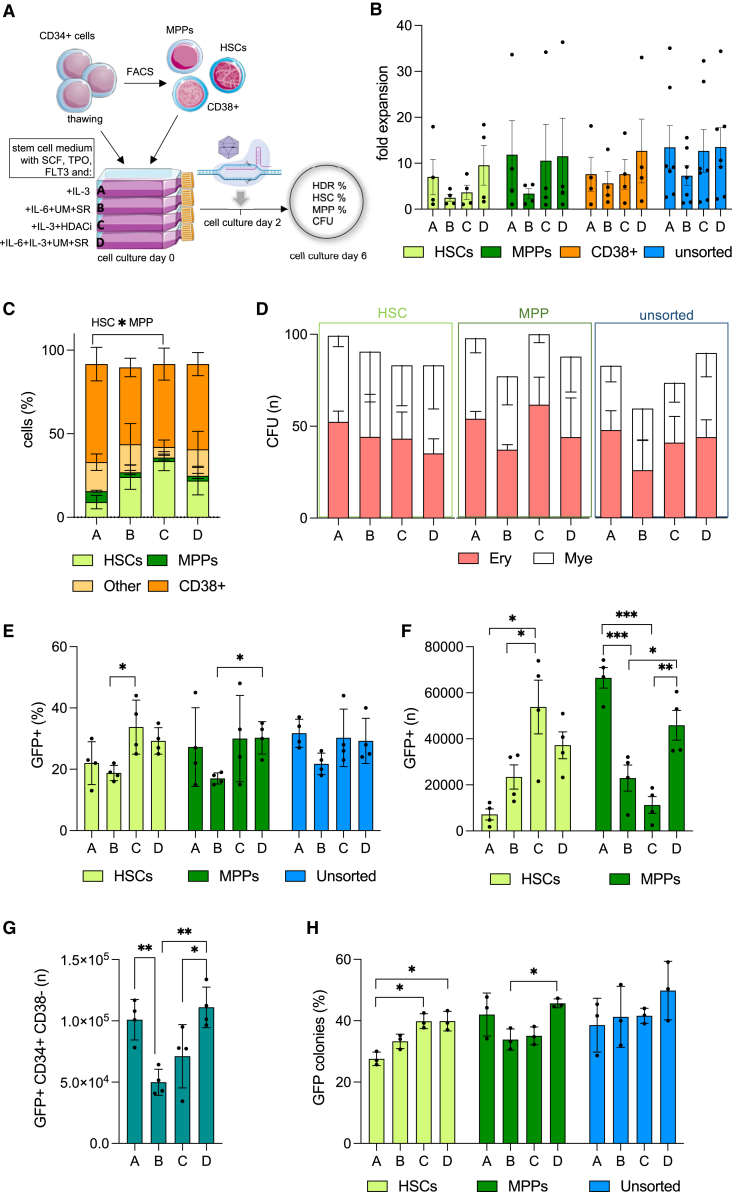
Evaluating the frequency of reporter gene knock-in and preservation of stemness in HSPC subpopulations (A) Schematic representation of the experimental procedure. CD34+ cells were thawed and FACS sorted into primitive HSCs (CD34+ CD38- CD90+ CD45RA-), MPPs (CD34+ CD38- CD90- CD45RA-) and CD38+ committed progenitors (CD34+ CD38+). HSCs, MPPs, CD38+, and CD34+ unsorted cells were then cultured into four different media (A–D) containing a distinct cocktail of cytokines and stem cell factors. After 2 days of culture, sorted and unsorted cells were gene-edited with the CRISPR-Cas9 platform and the AAV6 donor vector containing a PGK-GFP reporter cassette. After 4 additional days of culture, cells were harvested and analyzed. (B) Expansion rate (expressed as fold increase) of the different populations after 6 days of culture post thawing compared with day 1. (C) Phenotyping of the sorted HSC population at day 6 of culture. The plot shows the percentage of cells in the HSC-sorted population that has retained a HSC phenotype after cell culture, or has differentiated into MPPs, other CD38- progenitors (Other: CD34+ CD38- CD90-/low CD45RA+; this population may include LMPPs and MLPs), or to CD38+ committed progenitors (CD34+ CD38+), when cultured in the four different media. (D) Plots representing the number of white and red colonies formed in semi-solid culture media by sorted and gene-edited HSC and MPP populations and unsorted CD34+ cells cultured in the different media. (E) Percentage of cells harboring a GFP reporter cassette knocked in in the *WAS* locus in the sorted HSC and MPP populations or unsorted CD34+ cells cultured in the different media. (F) Number of cells harboring a GFP reporter cassette knocked in in the *WAS* locus in the sorted HSC and MPP populations cultured in the different media. (G) Number of edited repopulating CD34+ CD38- progenitors detected in the unsorted CD34+ samples cultured in the four different media. (H) Plots representing the percentage of GFP-positive colonies formed in methylcellulose by sorted and edited HSC and MPP populations or unsorted and edited CD34+ HSPCs cultured in the different media. HSC, hematopoietic stem cell; LMPP, lympho-myeloid primed progenitor; MLP, multi-lymphoid progenitor; MPP, multipotent progenitor. Data in Figure 2 are presented as mean ± SD, with n = 4 biological replicates in all panels except for (H), where n = 3. p values were calculated using one-way ANOVA with Tukey’s comparison test (∗p < 0.05; ∗∗p < 0.01; ∗∗∗p < 0.005; ∗∗∗∗p < 0.001; no asterisk = nonsignificant).

To accurately estimate rates of gene editing and preservation of stem cell phenotypes in different subpopulations of stem and progenitor cells, HSCs, MPPs, and more committed progenitors (CD38+; CD34+ CD38+ cells) were sorted from PB-derived CD34+ HSPCs immediately after cell thawing (gating strategy in Figure S2A) and placed in the four different culture media under investigation. Two days post sorting the three populations, as well as unsorted CD34^+^ cells, were edited by delivery of the Cas9/gRNA RNP and the AAV-PGK-GFP donor vector and rates of targeted integration, as well as the frequency of HSCs and MPPs in the culture, were determined 4 days after editing, for a total of 6 days of cell culture (Figure 2A). This timeline was purposely chosen to mimic a conventional HSPC gene therapy manufacturing protocol, which is usually 4–6 days long.

At day 6 of cell culture, cells cultured in IL-3-containing media (A, C, D) showed the highest fold of expansion in MPPs, CD38+, and CD34+ unsorted cells, with IL-6-containing medium B consistently yielding lower cell numbers (Figure 2B); however, the addition of HDACi restrained the proliferative effect of IL-3 specifically in HSCs, at levels similar to the medium B supplemented with IL-6, SR-1, and UM171. We then assessed preservation of the stemness phenotype and/or promotion of differentiation in HSCs sorted and cultured in the four medium compositions. Medium C showed the highest ability to maintain a stemness phenotype in HSCs, with an average of 33.6% of HSCs still showing surface labeling indicative of primitive HSCs (CD34+ CD38- CD90+ CD45RA-) 6 days post thawing, highlighting the ability of HDACi to preserve HSCs in culture. Given that the post culture cell numbers for medium C were equal to or lower than those observed for the other media (Figure 2B), we speculate that the enhancement in the HSC fraction mediated by medium C could be ascribed to stem cell preservation rather than their expansion. While HSCs cultured in medium B and D contained a slightly lower, although not significantly different, fraction of cells displaying a HSC phenotype compared with the best-performing medium (average 24% and 22%, respectively), sorted HSCs grown in medium A showed the most pronounced loss of stemness, with a >3-fold decrease in primitive HSCs compared with medium C (9.1%) (Figure 2C), confirming the results obtained previously. The HSC-preserving effect exerted by medium B and C was specific to the primitive stem cell pool, as no significant differences with the other media were observed in the MPP fraction; however, we did notice a favorable trend for media A and D in the preservation of MPPs after 6 days of culture, as already observed in Figure 1 (Figure S2B). Of note, this trend was already visible at day 2 post culture (gene editing day), when the frequency of HSCs halved in the HSC-sorted population cultured in medium A, further highlighting the strong stem cell differentiation signals induced by IL-3 already at early culture time points (Figure S2C). Similar to day 6, sorted HSCs cultured in medium C showed the highest phenotype preservation (average 88% of HSCs), followed by media B and D (average 77% of HSCs); again, no significant differences among media were observed in the phenotype preservation of MPPs and CD38+ cells (Figure S2C). The four populations were plated into methylcellulose cultures and were able to give rise to comparable proportions of myeloid and erythroid colonies with no skewing or significant difference in the colony-forming capacity among media (Figure 2D).

HDR-mediated knock-in of the reporter cassette was detected in all cell populations using all media combinations, with an overall comparable number of GFP-expressing cells in IL-3-supplemented media (Figure 2E). Medium C showed the highest levels of knock-in in HSCs followed by medium D, with IL-6-only medium B yielding overall a lower frequency of GFP-expressing cells (Figure 2E). No significant differences in the frequency of knock-in among different culture conditions was observed in unsorted CD34+ cells, highlighting the importance of studying the effects of *ex vivo* manipulation in carefully selected populations rather than retrospectively in cultured bulk CD34+ cells. Medium C yielded the highest number of edited cells displaying an HSC phenotype (CD34+CD38-CD90+CD45RA-) within the HSC-sorted population at culture day 6 followed by medium D, while both media A and D performed best in the MPP-sorted population (Figure 2F). All IL-3-containing media mediated efficient knock-in of the PGK-GFP cassette in unsorted CD34+ cells, yielding similar numbers of GFP+ cells, significantly exceeding the amount obtained with medium B (Figure S2D). Overall, when looking at the number of cells within the CD34+ CD38- population in unsorted CD34+ cells, which contains both HSCs, MPPs, and other progenitor types (such as the lymphoid-myeloid primed multi-potential progenitors [LMPPs] and the multi-lymphoid progenitors [LMPs]) and has been shown to be the major contributor of long-term engraftment in immunodeficient mice when transplanting edited HSPCs,^11^^,^^24^ medium D outperformed all the other culture conditions, providing the highest number of gene-edited, GFP-expressing bone marrow repopulating cells after 6 days of *ex vivo* culture (Figure 2G). HSC-, MPP-sorted and unsorted CD34+ cells yielded the highest output of GFP+ colonies in methylcellulose when cultured in medium D, demonstrating superior levels of knock-in in clonogenic progenitors when cells are manipulated *ex vivo* in presence of IL-3, IL-6, and stem cell agonists (Figure 2H).

### *In vivo* repopulating potential of HSPCs gene-edited in optimized cell culture conditions

**Figure 3:**
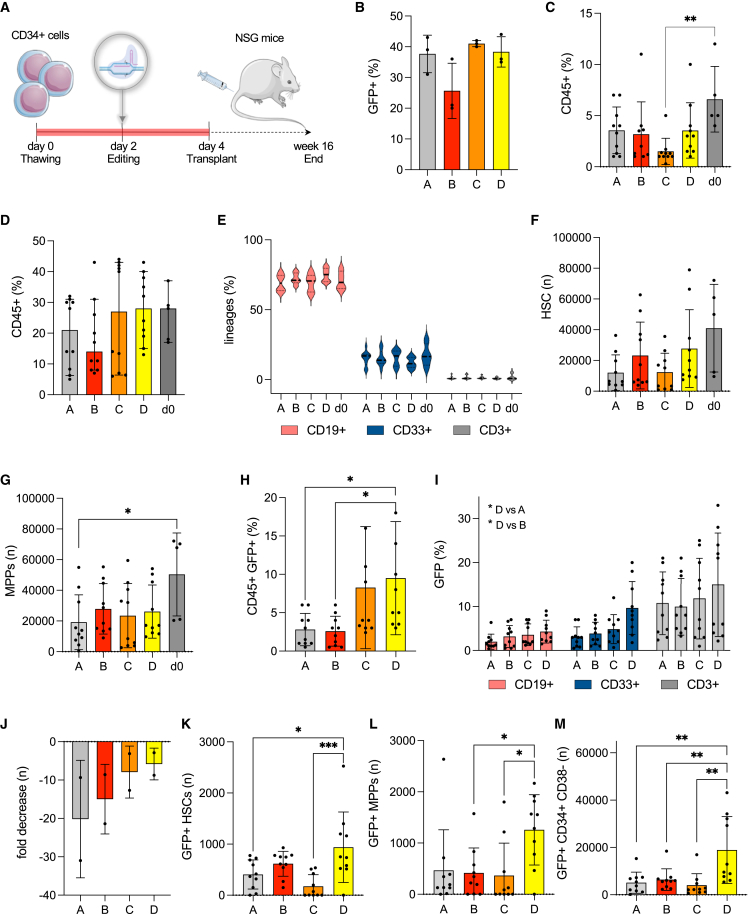
Evaluating the frequency of gene knock-in and hematopoietic reconstitution by gene-edited HSPCs *in vivo* A) Schematic representation of the experimental procedure. CD34+ HSPCs from three different healthy donors were thawed, cultured in media (A–D) and gene-edited at day 2. Two days later, cells were harvested and transplanted into sub-lethally irradiated NSG mice. Twelve to 16 weeks after transplantation, the experiment was terminated and the mice analyzed to detect human cell engraftment. (B) Rates of targeted integration (GFP+ cells) achieved *in vitro* in HSPCs pre-transplant. (C) Engraftment of human cells (CD45+) in the PB of NSG mice at 8 weeks post-transplant and (D) in the BM at 12–16 weeks post-transplant. (E) percentages of B (CD19+), myeloid (CD33+), and T (CD3+) human CD45+ cells retrieved from the BM of transplanted mice at termination. F) Number of human HSCs (CD34+ CD38- CD90+ CD45RA-) and (G) MPPs (CD34+ CD38- CD90- CD45RA-) detected in the BM of transplanted mice. (H) Percentage of human cells with a GFP reporter gene knocked in in the WAS locus (CD45+ GFP+) in the BM of experimental mice at 12–16 weeks post-transplant, respectively. (H) Percentage of human cells with a GFP reporter gene knocked in in the *WAS* locus (CD45+ GFP+) in the BM of experimental mice at 12–16 weeks post-transplant, respectively. (I) Percentage of human cells with a GFP reporter gene knocked in the *WAS* locus (CD45+ GFP+) in B (CD19+), myeloid (CD33+), and T cells (CD3+) retrieved from the BM of experimental mice at 12–16 weeks post-transplant. (J) Measurement of the reduction in the percentage of GFP-expressing cells (expressed as fold decrease) in the BM of mice at 12–16 weeks post-transplant compared with pre-transplant. (K) Number of GFP-expressing HSCs, (L) MPPs, and (M) CD34+ CD38− cells in the BM of mice at 12–16 weeks post-transplant. BM, bone marrow; HSC, hematopoietic stem cell; MPP, multipotent progenitor; PB, peripheral blood. Data in Figure 3 are presented as mean ± SD, with n = 10 mice transplanted with HSPCs from two different biological donors in all panels except for (B) where n = 3 biological replicates. p values were calculated using one-way ANOVA with Tukey’s comparison test, except for (I), where a two-way ANOVA with Bonferroni post-test was used (∗p < 0.05; ∗∗p < 0.01; ∗∗∗p < 0.005; ∗∗∗∗p < 0.001; no asterisk = nonsignificant).

We next evaluated whether HSPCs cultured and manipulated in the four different media retain their capacity to repopulate the bone marrow and differentiate into all the hematopoietic lineages *in vivo*. For this set of experiments, we decided to transplant only unsorted CD34+ HSPCs, as this is the main population currently used for gene therapy and gene editing clinical application and product manufacturing. CD34+ HSPCs from three different healthy donors were thawed, placed in culture in media A–D and edited following our standard protocol; at day 4 of culture, 0.5 × 10^6^ cells were transplanted into 8-week-old sub-lethally irradiated immunodeficient non-obese diabetic (NOD)-SCID Il2rg^−/−^ (NSG) mice, alongside NSG mice transplanted with thawed and uncultured HSPCs (d0) as a control (Figure 3A). An average targeted integration of the PGK-GFP cassette in 25%–40% of the cells was detected in all the samples *in vitro*, with medium B resulting in the lowest knock-in frequency in 2 of 3 donor sources, a trend that was visible even at later culture days (Figures 3B and S3A). Short-term human engraftment was measured in the PB of mice at week 8 after transplantation, with the highest chimerism observed in mice transplanted with unmanipulated, uncultured HSPCs, and the lowest with cells cultured in medium C (Figure 3C). At 14 weeks post-transplant, medium C and D showed a higher frequency of engraftment (measured as percentage of human CD45+ cells) in the bone marrow (BM) and PB of mice compared with the other media and, importantly, with similar levels compared with unmanipulated controls (Figures 3D and S3B). This difference was particularly evident when analyzing animals transplanted with different cell donor sources separately to eliminate the background noise deriving from donor-to-donor variability (Figure S2C). Mice transplanted with CD34+ cells retrieved from donor 3 displayed extremely low levels of engraftment in all experimental and control groups, possibly due to the CD34+ cell donor source, as even mice injected with uncultured and unmanipulated cells (d0) showed minimal engraftment; therefore, data from these animals were discarded from all subsequent analyses for the impossibility to study less abundant populations, such as HSCs and MPPs (Figure S3D). Cells derived from all four different experimental groups were able to differentiate into mature CD19+ B cells, CD33+ myeloid cells, and CD3+ T cell precursors without lineage skewing compared with unmanipulated cells (Figure 3E), indicating preserved functionality of cultured and gene-edited HSPCs. We then investigated the composition of the CD45+ engrafted human cell population in the BM of transplanted mice. We observed a higher number of CD34+ CD38- CD90+ CD45RA- HSCs in media B and D compared with the others, although with an overall lower frequency compared with non-cultured controls, as expected (Figures 3F and S3E); of note, among the two best-performing media, medium D appeared to mediate the highest HSC engraftment rate in the BM when transplanting CD34+ derived from donor 1 (Figure S3B). The number of engrafted MPPs was similar among the media formulations explored (Figure 3G), with, however, medium B and D resulting in the highest amount of MPPs found in the BM of transplanted mice for donor 1 (Figure S3B). Contrary to observations for HSCs *in vitro* (Figures 2E–2H), mice transplanted with HSPCs cultured in medium C yielded the lowest number of engrafted HSCs in the BM.

When examining the rate of targeted integration of the PGK-GFP reporter cassette in the *WAS* locus in human engrafted CD45+ cells in the BM of experimental mice, an average of 8.2% and 9.5% of GFP-expressing cells was detected in the medium C and D groups, respectively (Figure 3H), with a more than 2-fold increase compared with the other media analyzed. When looking at the percentage of mature human B, myeloid, and T cells harboring a correct reporter cassette in the *WAS* locus, medium D outcompeted the other conditions, with up to 20%, 9%, and 33% CD45+ GFP+ cells, respectively (Figure 3I). Although we still observed a marked drop in the rate of HDR-mediated knock-in post-transplantation *in vivo* compared with the *in vitro* counterpart, the reduction was much less evident when using the two best-performing media, which showed less than 10-fold decrease on average in the frequency of gene-edited cells post-transplantation compared with IL-3 only or IL-6 only media (Figure 3J). However, when carefully examining HSPC subpopulations expressing GFP, medium D yielded the greatest number of edited human HSCs and MPPs engrafted in the BM, and an overall significant increase in the number of GFP-expressing CD34+ CD38- long-term repopulating cells compared with medium C and all the other conditions tested (Figures 3K–3M and S3F).

## Discussion

The data presented here identify the need of a fine-tuned balance between cues that promote cell proliferation and preservation of stemness and repopulating potential for the *ex vivo* manipulation of HSPCs for therapeutic purposes. *Ex vivo* gene therapy approaches for blood disorders rely on the extended culture of HSPCs *in vitro*, which is required to achieve genetic engineering of target cells while expanding them, in order to attain sufficient cell^43^ numbers for a beneficial transplant into the patient. Several studies have demonstrated successful culture of adult and cord-blood (CB)-derived HSPCs, although prolonged *in vitro* culture has been associated with a decrease in their *in vivo* engrafting potential and lineage skewing upon hematopoietic reconstitution.^22^^,^^23^^,^^24^^,^^34^^,^^44^^,^^45^ The diminished repopulating capacity of *ex vivo* cultured HSPCs can be partially explained by deregulation of stemness-related signaling pathways, such as Wnt or Notch, increased proliferation cues and decreased expression of molecules involved in HSC homing under expansion conditions, resulting in reduced engraftment and promotion of stem cell differentiation.^21^^,^^22^^,^^46^ Genetic manipulation with viral vectors, and particularly with the gene editing machinery, can lead to an additive and even more pronounced defect in the HSC compartment, by triggering a p53-mediated response that can further induce cell differentiation or apoptosis.^12^^,^^47^ From a clinical perspective, this progressive reduction in the hematopoietic potential of ex vivo-engineered HSPCs can result in low levels of chimerism post-transplant as well as delayed immune recovery after myeloablation. As such, defining new culture methods that allow correct culture of HSPCs while preserving their stemness features is fundamental for the therapeutic success of gene therapy approaches. Recently, the introduction of protocols using UM171 and SR-1 to culture HSPCs has achieved the successful *in vitro* expansion of repopulating cells that supported durable engraftment in immunodeficient mice,^14^^,^^24^ partially overcoming the drawbacks of prolonged culture.

In this regard, here we have tested the use of different culture conditions in the context of genetic correction via gene editing. While protocols to mediate efficient HDR-mediated gene editing in HSPCs have already been established and extensively used in the labs, here we have focused our efforts on dissecting the contribution of the culture medium composition on the preservation of more primitive progenitors (HSCs and MPPs) and their efficient gene editing. Indeed, gene knock-in via CRISPR/Cas entails a further critical limiting factor to the ones mentioned above, that is the relatively low numbers of HDR-corrected HSCs obtained during the manufacturing process, which may lead to graft failure and lack of therapeutic benefit when infused into patients, especially those suffering from conditions that require high levels of chimerism to be treated. Therefore, reaching high rates of genetic correction in BM repopulating cells is also a key aspect to consider when choosing the right HSPCs culture protocol. In light of these considerations, our data show that a medium containing IL-3, which stimulates cell proliferation, could be favorably used for HSPC gene editing to obtain a high number of HDR-corrected cells. However, the pronounced differentiation of more primitive HSCs induced by IL-3 influences their long-term BM repopulating ability, resulting in lower engraftment of HSCs once transplanted *in vivo*. Indeed, despite rates of BM engraftment similar to those achieved when infusing HSPCs cultured in the other media, the number of HSCs found post-transplant was one of the lowest among the conditions tested. The same holds true when looking at engrafted cells with a correct GFP reporter cassette integrated in the *WAS* locus; indeed, medium A showed the highest drop in the percentage of GFP+ cells when comparing pre- and post-transplant gene editing rates, suggesting that while promoting proliferation and activation of the HDR pathway, it also induces loss of BM repopulating cells, likely due to cell differentiation. On the contrary, IL-6-, SR-1- and UM171-supplemented medium B better preserved the fraction of HSCs when cultured *ex vivo*, both *in vitro* and *in vivo*, at the expense of cell expansion and knock-in rates, despite the fact that these stem cell agonists have been shown to promote proliferation of CB-derived HSPCs.^25^^,^^26^ Indeed, medium B yielded persistently lower rates of GFP+ cells *in vitro* and *in vivo* compared with other medium formulations, even though it mediated the preservation of high numbers of GFP+ HSCs in the BM of transplanted mice.

We therefore reasoned that optimizing culture conditions by integrating the advantages of the two media could lead to an optimal balance between proliferation and stem cell preservation. We indeed showed that the differentiation program activated by IL-3 can be counteracted by the addition of stem cell preserving compounds, such as HDACi, IL-6, SR-1, and UM171. Exposure of HSPCs to HDACi when cultured in medium C preserved a primitive stem cell phenotype *in vitro*, and through the proliferative activity mediated by IL-3 contained in the medium, allowed achievement of high rates of HDR-mediated reporter gene knockin (see Figures 2C and 2F). This is in line with previous reports using HDACi in CB HSPC cultures, which showed that inhibition of HDAC1, -3 and -5 proteins favors retention and/or induction of a primitive stem cell program, promotion of self-renewal and an increased homing capacity in CD34+ CD38- CD90+ CD45RA- HSCs, while the concomitant presence of cytokines favored their expansion *in vitro*.^37^^,^^48^ However, while cells cultured and edited in medium C engrafted at high levels *in vivo* with one of the highest proportions of CD45+ GFP-expressing cells among the conditions tested, the number of HSCs and GFP+ HSCs found in the BM post-transplantation was the lowest observed in our experimental settings. One possible explanation is that the program installed by HDACi in HSCs promotes the quiescence of HSCs once engrafted in the BM and in the absence of proliferation-inducing cytokines used for the *ex vivo* culture; indeed, when HDACi are added to the HSPC culture without IL-3, cells are unable to proliferate and undergo HDR-mediated GFP knock-in despite showing high numbers of CD38- CD90+ cells (A.C. and G.S., unpublished data and Chaurasia et al.^37^), indicating that they promote via epigenetic remodeling a stem cell-defining gene expression program that includes quiescence. This is further backed up by the limited cell expansion observed when sorted HSCs are cultured in medium C, an effect that is specific to that population and not detected in MPPs and unsorted CD34+ or CD38+ cells, and by the delayed repopulating capacity of HSCs cultured in medium C when transplanted into mice (see Figures 3C and S3B for a comparison between engraftment rates at 8 and 12–16 weeks post-transplantation). Another possibility is that HDACi’s stem cell agonist effect is induced only transiently during the *ex vivo* culture, especially in adult HSPCs that have a reduced plasticity compared with CB-derived ones. In support of this, previous studies conducted in CB HSCs have reported a positive effect on the *in vivo* repopulating capacity of HDACi-treated HSCs only when transplanted into immunodeficient mice after a very short culture period (<24 h),^37^ while a prolonged period of incubation resulted in loss of such an effect.^49^

The addition of IL-3, IL-6, SR-1, and UM171 to the HSPC medium is the most compelling strategy tested here, as it represents the best compromise between high editing efficiency and preservation of BM repopulating cells. Indeed, IL-3 contained in medium D promoted cell proliferation at sufficient levels to achieve one of the highest rates of HDR-mediated GFP knock-in and cell expansion among the media tested (see Figure 2E). On the contrary, the presence of IL-6 and stem cell agonists likely counterbalanced the differentiation signals promoted by IL-3 and supported the preservation of HSCs in culture at levels comparable to the current state-of-the-art media used in preclinical HSPC gene editing studies (medium B). The combination of these effects resulted in a higher amount of both HSCs and MPPs that were correctly gene-edited, which also translates into the highest number of GFP+ CD34+ CD38- repopulating HSPCs among the conditions tested (see Figures 3K–3M), although serial transplantation studies would ultimately be required to confirm the superiority of this culture condition in mediating gene editing in long-term repopulating cells. The dual effect on both HSCs and MPPs is of paramount importance considering that, for a therapeutically successful *ex vivo* gene therapy application, both populations must be corrected and infused into the patient, as the latter provides for a fast, short-term hematopoiesis within the first months after the transplantation, ensuring rapid immunological reconstitution.^5^^,^^49^ Hence, culturing HSPCs in a medium that promotes maintenance and editing of both populations represents an undisputed advantage for therapeutic success and a step forward from existing HSPC gene editing protocols. In conclusion, our data provide a careful dissection of the effects that currently used media exert on *ex vivo* cultured HSPCs and suggest an improved medium formulation for gene editing protocols toward the enhanced clinical translation of HDR-based therapies.

## Materials and methods

### Human CD34+ HSPC culture

PB samples mobilized through a combination of Plerixafor (Mozobil) and GCS-F (Neupogen) from male healthy donors were isolated under written informed consent. Within 24 h of scheduled apheresis, CD34*+* HSPCs were purified using CD34+ Microbead kit (Miltenyi Biotec, UK) according to the manufacturer’s protocol. Purity was assessed by staining with anti-human CD34 BV 421 antibody (clone 561, BioLegend, USA). For long-term storage, the cells were frozen in CryoStor cell cryopreservation media (Sigma-Aldrich). After thawing, cells were cultured in StemSpan ACF (StemCell Technologies, USA) supplemented with hSCF (100 ng/mL, Peprotech, UK), hTPO (100 ng/mL, Peprotech), hFlt3-ligand (100 ng/mL, Peprotech), and a combination of either hIL-3 or hIL-6 (30 ng/mL, PeproTech, UK), SR-1 (1 μM, R&D Systems), UM171 (35 nM, STEMCELL Technologies) and the HDAC inhibitor ScriptAid (1 μM, Sigma) at a density of 2.5 × 10^5^ cells/mL. Cells were incubated at 37°C/5% CO_2_ for 2 days prior to electroporation.

### Electroporation and transduction

After 2 days in culture, CD34+ HSPCs were electroporated using Neon Transfection kit (ThermoFisher Scientific). Briefly, 0.2 × 10^6^ cells were centrifuged and re-suspended in ribonucleoprotein (RNP) complex. The RNP was made by incubating a chemically modified (with 2-O-methyl-3′-phosphorothioate at the three terminal positions at both 5′ and -3′ ends; Synthego, USA^50^) single gRNA targeting *WAS,*^3^ with the Alt-R S.p. HiFi Cas9 protein^51^ (Integrated DNA Technologies, USA) at a molar ratio of 1.5:1 at 37°C for 15 min. The condition for electroporation was 1600 V, 10 m, three pulses. Following electroporation, cells were seeded at concentration of 1 × 10^6^ cells per mL and incubated at 37°C for 15 min after which AAV6 at 25,000 MOI (Vector genomes/cell) was added and incubated.

### Editing of CD34+ HSPC subsets

At day 0, CD34+ HSPCs were thawed and stained with the following antibody panel: CD34 BV 421, CD38 APC-Cy7 (clone HIT2, BioLegend), CD90 PE-Cy7 (clone 5E10, BioLegend), and CD45RA APC (clone HI100, BioLegend). The cells were then FACS sorted into different subsets including CD38+ cells, HSCs and multipotent progenitors (MPPs). Unsorted cells were used as an additional cell population. After 2 days, both sorted and unsorted cells were electroporated and transduced with AAV6_PGK-GFP. At days 5 and 12 post editing, the cells were stained with the above antibody panels to determine the percentage of HSCs and MPPs as well as evaluate GFP expression by flow cytometry. Cell yields were calculated by adding 10 μL of Count Bright Absolute Counting Beads (ThermoFisher Scientific), following the manufacturer’s protocol.

### Detection of INDELs

After 4 days of editing, genomic DNA was extracted using DNeasy Blood and Tissue extraction kit (Qiagen) and the region of the RNP cut site was PCR amplified. The sequencing results were processed through TIDE software (www.tide.nki.nl) to determine INDEL frequency.

### Methylcellulose CFU assay

The colony-forming unit (CFU) assay was performed by seeding 500 cells in six-well plates containing MethoCult Enriched (StemCell Technologies) after 4 days of editing. After 14 days of incubation at 37°C/5% CO_2_, different types of colonies including CFU-Erythroid (E), CFU-Macrophage (M), CFU-Granulocyte (G), CFU-GM, and CFU-GEM were counted based on their morphological appearance.

### Transplantation of genome-edited CD34+ HSPCs in NSG mice

NSG adult female mice (6–8 weeks old) were purchased from Charles River and were sub-lethally irradiated (3 Gy) 24 h prior to transplantation. CD34+ HSPCs were thawed and cultured. At day 4 of culture (2 days after editing), 0.5 × 10^6^ viable cells/mouse were injected via tail vein into NSG mice with a 27-gauge × 0.5-inch needle. The same cell number per mouse was used for all experimental conditions tested. After 8 and 14 weeks post-transplantation, mice PB from the tail vein was lysed with 1× RBC lysis buffer (ThermoFisher Scientific) and stained with anti-human CD45 APC antibody (clone HI30, BioLegend) to evaluate human CD45+ engraftment by flow cytometry. At 14–16 weeks post-transplantation, level of human engraftment and lineage composition were determined in the BM and PB. Briefly, BM cells were harvested by flushing tibiae and femurs with 1× PBS and passing through a 40-μm strainer. Mononuclear cells were blocked with Fc blocking solution (BioLegend) and stained with the following antibody panels; CD45 BV421, CD19 PerCp Cy5.5 (clone HIB19, BioLegend), CD33 FITC (clone P67.6, BD Bioscience), and CD3 PE (clone OKT3, BioLegend) for flow cytometry analysis. To determine human stem cell composition within BM, cells were stained with the following antibody panels: CD45 BV421, CD34 FITC, CD38 APC-Cy7, CD90 PE-Cy7, and CD45RA PerCP Cy5.5.

### Flow cytometry analysis

BD FACSAria II (BD Bioscience) instrument was used for cell sorting of CD34+ HSPCs. For all flow cytometry analyses, a BD LSRII instrument (BD Bioscience) was used. Antibody dilutions used for staining was 1 in 100. For data anlaysis, FlowJo v10 software (FlowJo LLC, USA) was used.

### Digital droplet PCR analysis

Digital droplet PCR (ddPCR) was performed to measure the frequency of PGK-GFP integration in the edited samples. Briefly, genomic DNA was extracted 4 days post editing using DNeasy Blood and Tissue extraction kit (Qiagen, UK). In a total volume of 22 μL, 20 ng of genomic DNA was combined with 10 μM each of target primer and FAM probe mix, 10 μM each of reference primer and HEX probe mix, 1× ddPCR Supermix probe without dUTP (Bio-Rad, UK) and nuclease-free water. The individual droplets were generated using QX100 Droplet Generator (Bio-Rad) and subsequently amplified in a Bio-Rad PCR thermocycler. The optimized amplification steps were as follows: step 1, 95°C for 10 min; step 2 (49 cycles), 94°C for 1 min, 60°C for 30 s, 72°C for 2 min; and step 3, 98°C for 10 min. The Droplet Reader and QuantaSoft Software (both from Bio-Rad) were used to record and analyze the positive and negative fluorescence droplets according to the manufacturer’s guidelines (Bio-Rad). The percentage of integration was calculated as the ratio of FAM to HEX signal after normalization against the reference signal.

### Statistical analysis

All the experiments were carried out at least thrice independently. All data are presented as means ± SD. Animals of similar age, weight, and sex were grouped randomly, and the number of animals per group was 5–10. Statistical analyses were conducted using GraphPad Prism 9 (GraphPad Software Inc, USA). Parametric and nonparametric tests were used based on the population distribution (normally vs. non-normally distributed variables, respectively) and sample size. Specifics of each statistical test used can be found in the figure legends. For making multiple comparisons, we used one-way ANOVA with Tukey’s multiple comparisons test with one variable and two-way ANOVA with Bonferroni post-test in case of two variables. For comparing the average mean of two sample groups, we used the unpaired Student’s t test to reject the null hypothesis (p < 0.05). p < 0.05 was considered significant for all tests.

### Ethics and animal approval statement

For usage of human CD34+ HSPC from healthy donors, informed written consent was obtained in accordance with the Declaration of Helsinki and ethical approval from the Great Ormond Street Hospital for Children NHS Foundation Trust and the Institute of Child Health Research Ethics (08/H0713/87).

For experiments involving animals, mice were housed in a 12-h day-night cycle with controlled temperature and humidity. The ventilated cages had sterile bedding and everyday supply of sterile food and water in the animal barrier facility at University College London. Mice were bred and maintained in accordance with UK Home Office regulations, and experiments were conducted after approval by the University College London Animal Welfare and Ethical Review Body (project license 70/8241).

## Data availability

We declare that the data supporting the findings of this study are available within the paper and its supplementary information files or from the authors upon request.

## Supplemental information

Document S1. Figures S1–S4

Document S2. Article plus supplemental information
