# Supplementary material for: An improved medium formulation for efficient ex vivo gene editing, expansion and engraftment of hematopoietic stem and progenitor cells
Source: Mol Ther Methods Clin Dev. 2023 Feb 28;29:58–69. doi: 10.1016/j.omtm.2023.02.014 (PMC10025975; doi:10.1016/j.omtm.2023.02.014)
Supplement: Document S1. Figures S1–S4 [file mmc1.pdf]

OMTM, Volume *29*

Supplemental information

An improved medium formulation for efficient *ex vivo* gene editing, expansion and engraftment of hematopoietic stem and progenitor cells

Rajeev Rai, Asma Naseem, Winston Vetharoy, Zohar Steinberg, Adrian J. Thrasher, Giorgia Santilli, and Alessia Cavazza

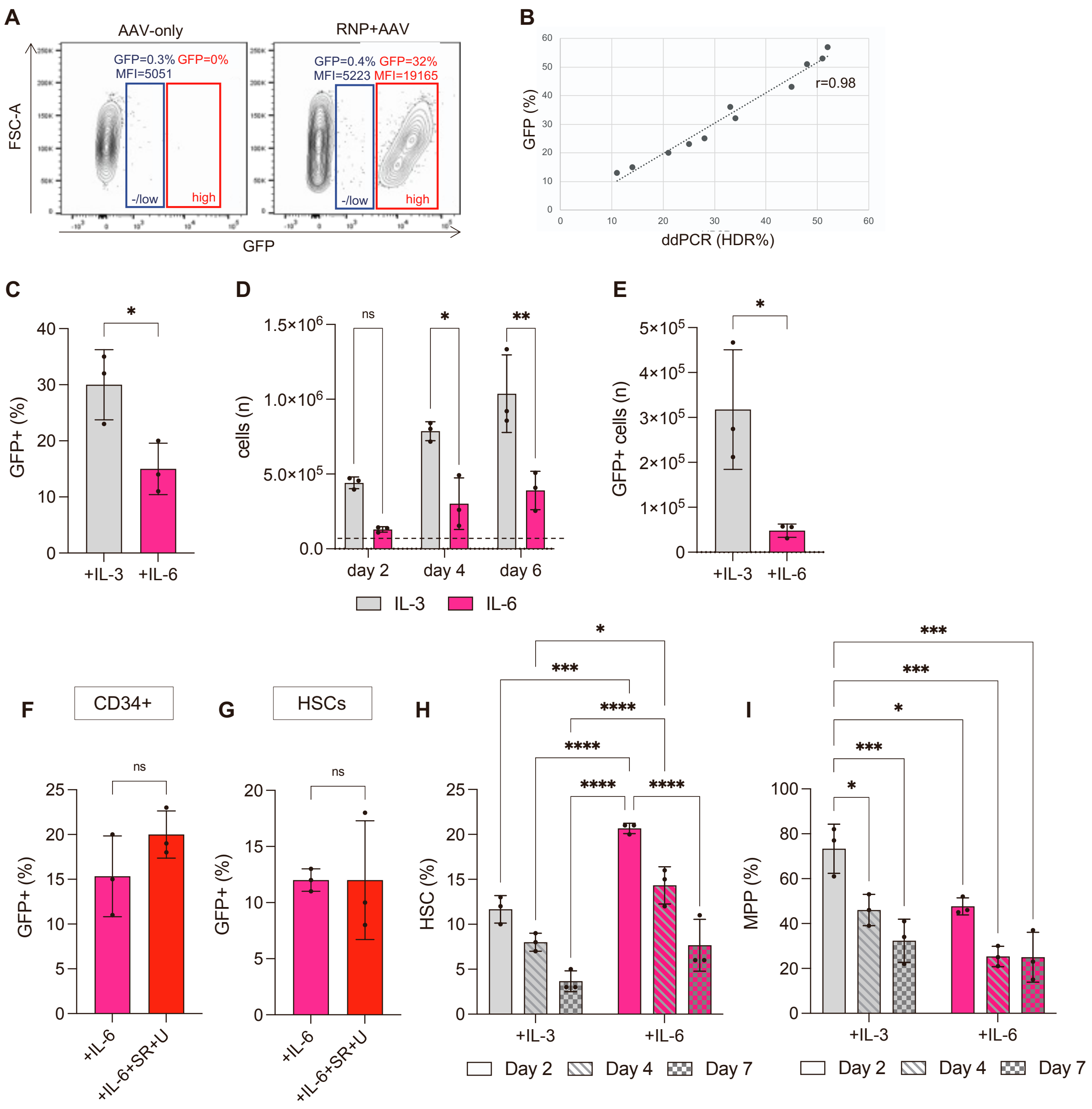

Figure S1. Comparison of *ex vivo* culture and manipulation of HSPCs grown in media supplemented with either IL-3 or IL-6. A) Analysis of GFP expression by flow cytometry in HSPCs transduced with AAV6 PGK-GFP (AAV-only) or CRISPR/cas9 gene-edited with an RNP targeting the WAS locus and transduced with the PGK-GFP cassette (RNP+AAV). GFP detection by flow cytometry is highly specific from day 2 post-editing, when gating only GFP^{high} cells. Indeed, the possible signal from episomal AAV as detected in AAV-only infected

HSPCs is minimal when gating on GFP^{-} or GFP^{low} cells and zero when gating on GFP^{high} cells, with 4-fold difference in the GFP MFI between GFP^{low} and GFP^{high} cells. **B)** ddPCR was performed to detect the integration of the PGK-GFP cassette in exon 1 of the *WAS* locus. Of note, being WAS on the X chromosome and being all the HSPC donor used in this study all of male origin, the frequency of monoallelic modification detected by ddPCR is a direct measure of the percentage of cells that have integrated a correct PGK-GFP cassette in the *WAS* locus (HDR%). Analysis of GFP expression by flow cytometry confirmed the results obtained by ddPCR with a Pearson correlation of 0.98. **C)** Frequency of HDR-mediated knock-in (GFP+) of a PGK-GFP reporter cassette at the *WAS* locus in CD34+ HSPCs cultured in IL-3- or IL-6-supplemented medium; **D)** Total number of cells retrieved after 2, 4 and 6 days of culture in the two media. The dotted line represents the starting cell number (100,000 cells)**; E)** Total number of edited cells retrieved in either medium after 6 days of culture; **F)** Frequency of HDR-mediated knock-in (GFP+) of a PGK-GFP reporter cassette at the *WAS* locus in CD34+ HSPCs and in **G)** sorted HSCs (CD34+CD38- CD90+ CD45RA- cells) when cultured in a IL-6- or IL-6+SR1+UM171-supplemented medium (medium B); **H)** Frequency of HSCs (CD34+CD38- CD90+ CD45RA- cells) and **I)** MPPs (CD34+CD38-CD90-CD45RA- cells) detected in the CD34+ bulk cultured in either medium 4 and 7 days after gene editing (6 and 9 days of culture, respectively). HDR: homology-directed repair, HSC: hematopoietic stem cell, MPP: multipotent progenitor.

Data in Figure S1 are presented as mean ± SD, with n=3 biological replicates, except for panel B where n=11. *P*-values were calculated using one-way ANOVA with Tukey's comparison test (panels H and I) or two-tailed unpaired Student's *t* test (panels C-G) (* p<0.05; ** p<0.01; *** p<0.005; **** p<0.001; no asterisk=non-significant).

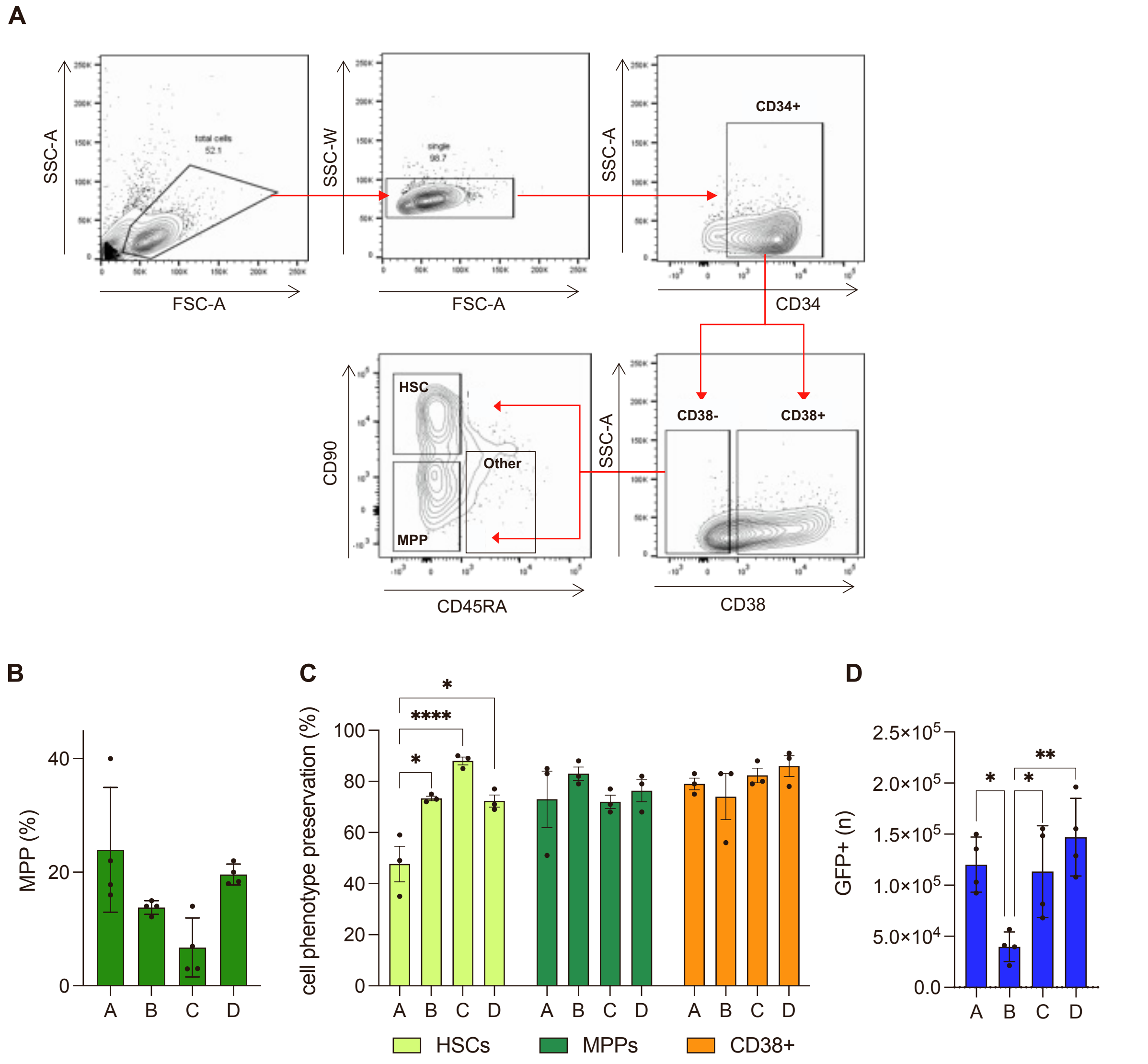

Figure S2. Evaluating the frequency of HSPC subpopulations *in vitro*. A) Representative gating strategy for the isolation/analysis of the different cell populations from CD34+ HSPCs by flow cytometry (HSCs: CD34+ CD38- CD90+ CD45RA-; MPP: CD34+ CD38- CD90- CD45RA-; Other CD38- progenitors: CD34+ CD38- CD90-/low CD45RA+; CD38+: CD34+ CD38+). **B)** Percentage of MPPs cells with a MPP phenotype after 6 days of culture in the 4 different media (A-D). **C)** Phenotyping of the sorted HSC, MPP and CD38+ cell populations at day 2 of culture. The plot shows the percentage of cells in the HSC, MPP or CD38+ sorted

population that have preserved their HSC, MPP, and CD38+ phenotype, respectively, after culture in the 4 different media. **D)** Number of cells harbouring a GFP reporter cassette knocked in in the *WAS* locus in the sorted HSC and MPP populations cultured in the different media. HSC: hematopoietic stem cell, MPP: multipotent progenitor.

Data in Figure S2 are presented as mean ± SD, with n=4 biological replicates, except for panel C where n=3 biological replicates. *P*-values in B were calculated using one-way ANOVA with Tukey's comparison test (* $p<0.05$; ** $p<0.01$; *** $p<0.005$; **** $p<0.001$; no asterisk=non-significant).

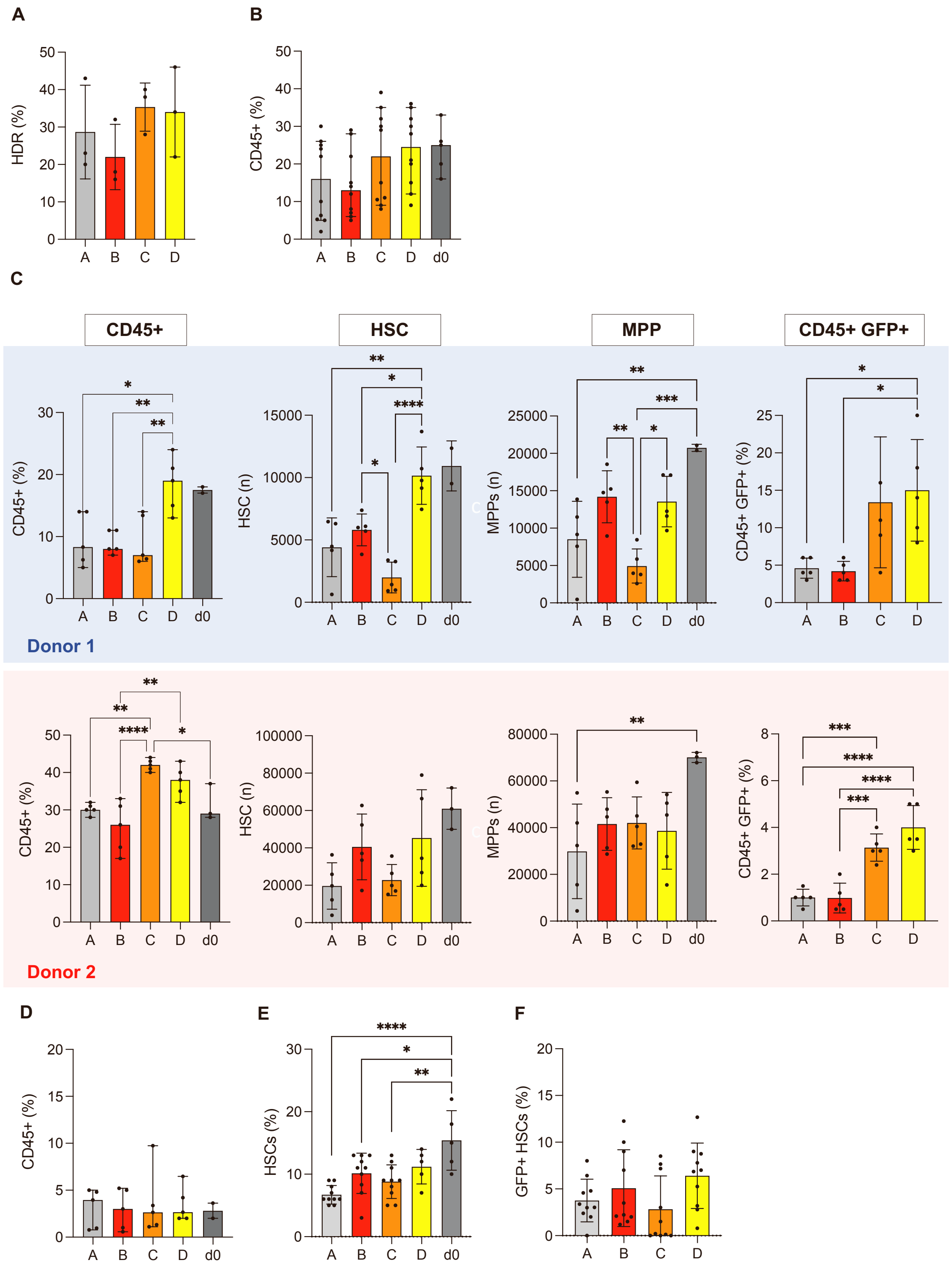
A
HDR (%)
B
CD45+ (%)
C
CD45+
HSC
MPP
CD45+ GFP+
CD45+ (%)
HSC (n)
MPPs (n)
CD45+ GFP+ (%)
Donor 1
Donor 2
D
CD45+ (%)
E
HSCs (%)
F
GFP+ HSCs (%)
A
B
C
D
d0

Figure S3. Evaluating the frequency of gene knock-in and hematopoietic reconstitution by gene-edited HSPCs *in vivo*. A) Rates of targeted integration achieved *in vitro* in HSPCs pre-transplant after 9 days of culture in the 4 different media (A-D); **B)** Engraftment of human cells (CD45+) in the PB of NSG mice at 8 weeks post-transplant per each medium condition **C)** Percentage of engrafted cells (CD45+), number of HSCs and MPPs, and percentage of GFP-expressing engrafted cells (CD45+GFP+) in the BM of transplanted mice, for each donor source considered in **Figure 3**; **D)** Engraftment rate in the BM of mice transplanted with HSPCs harvested from Donor 3. **E) Frequency** of human HSCs (CD34+CD38- CD90+ CD45RA-) and **F)** gene-edited (GFP+) HSCs detected in the BM of transplanted mice at week 14-16.

Data in Figure S3 are presented as mean ± SD, with n=10 mice transplanted with HSPCs from 2 different biological donors in all panels except for A where n=3 biological replicates, and D where n=5 mice transplanted with HSPCs from 1 biological donor. *P*-values were calculated using one-way ANOVA with Tukey's comparison test (* p<0.05; ** p<0.01; *** p<0.005; **** p<0.001; no asterisk=non-significant).

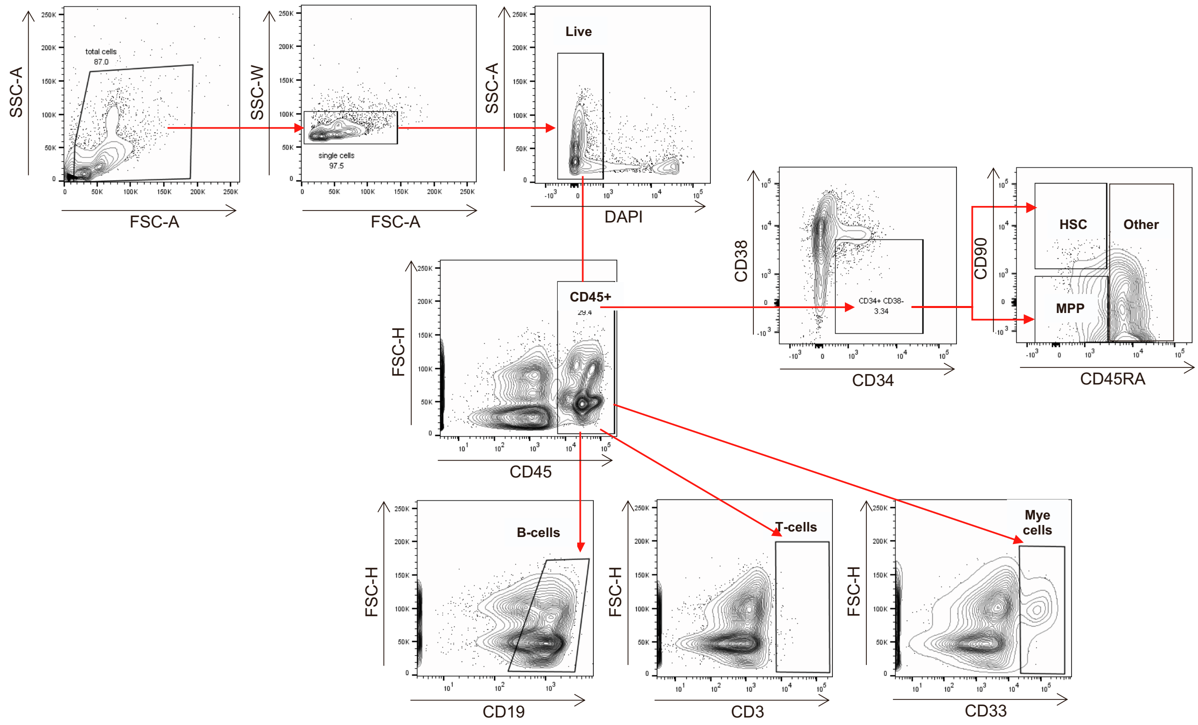

Figure S4. Representative gating strategy for the analysis of HSPC engraftment in vivo by flow cytometry.
